# The laparoscopic transperitoneal approach for irreducible inguinal hernias: Perioperative outcome in four patients

**DOI:** 10.4103/0972-9941.55104

**Published:** 2009

**Authors:** Rajan B Jagad, Jignesh Shah, Gulabbhai R Patel

**Affiliations:** Department of Surgery, New Civil Hospital and Government Medical College, Surat, Gujarat, India

**Keywords:** Irreducible hernia, laparoscopy, transperitoneal approach

## Abstract

**BACKGROUND:**

Incarceration and strangulation are the most feared complications of inguinal hernia. Till date, incarcerated hernias have traditionally been treated by conventional open repair. Reports are now available for the feasibility of laparoscopic repair of incarcerated inguinal hernia. Here, we described our experience with the transperitoneal approach for incarcerated hernias.

**MATERIALS AND METHODS:**

Between January 2008 and May 2008, four patients were presented with a history of irreducible hernia, abdominal distention and vomiting. All the patients had right-sided inguinal hernia. Reductions of the hernia contents were not possible in any patient. The patients were treated on emergency basis with laparoscopic transabdominal preperitoneal hernia repair. Retrospective analyses of all the patients were done.

**RESULTS:**

Reduction of the bowel was achieved in all but one patient, who required the division of the internal ring on lateral side. Transperitoneal mesh repair was performed. No major complications were encountered. One patient developed seroma formation that was treated conservatively.

**CONCLUSION:**

Laparoscopic transperitoneal approach has the advantage of observation of the hernia content for a longer period of time. The division of the internal ring can be done under direct vision. Other intra-abdominal pathology and opposite side hernia can be diagnosed and treated at the same time..

## INTRODUCTION

Laparoscopic treatment of inguinal hernia was first described by Schultz *et al*. in 1990.[1] Since then the laparoscopic approach has spread rapidly throughout the world. Compared with the conventional technique, the laparoscopic approach had been shown to reduce postoperative pain and improve the quality of life.[2,3] The most feared complications of inguinal hernias are incarceration, obstruction and strangulation. The probability of incarceration hernia varies from 0.29 to 2.9%.[4] Gangrene of the gut occurs in about 10–15% of hernia incarcerations.[5,6] Till recent time, surgical management of incarcerated groin hernia was done by open surgery. The laparoscopic approach was considered relative contraindication for incarcerated groin hernia. Recent data suggest that the laparoscopic approach is a feasible option for the management of incarcerated groin hernia.[7–9]

Here, we described our experience with emergency surgery for incarcerated hernia and review of the literature.

## MATERIALS AND METHODS

Between January and May 2008, four consecutive patients with a mean age of 42 years (range: 26–59) were admitted to our hospital on emergency basis with complaint of incarcerated hernia. All patients were males. The contents of the hernia could not be returned to the abdominal cavity by manipulation. In all four patients, preoperative abdominal x-ray showed increased gas shadow in the small bowel. In all patients, incarcerated hernias were treated laparoscopically.

## SURGICAL TECHNIQUE AND RESULTS

**Figure 1:**
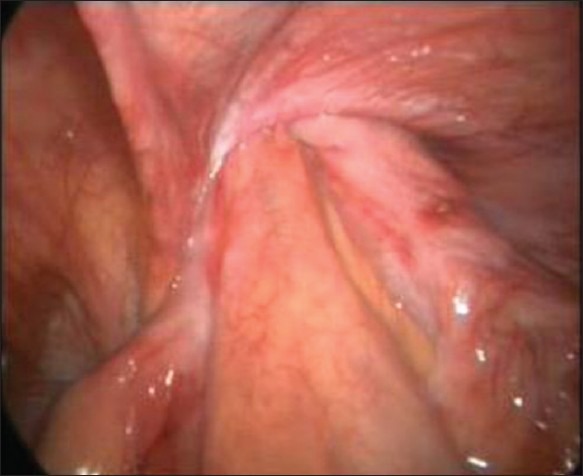
Irreducible herniation of the small bowel through the deep ring

**Figure 2:**
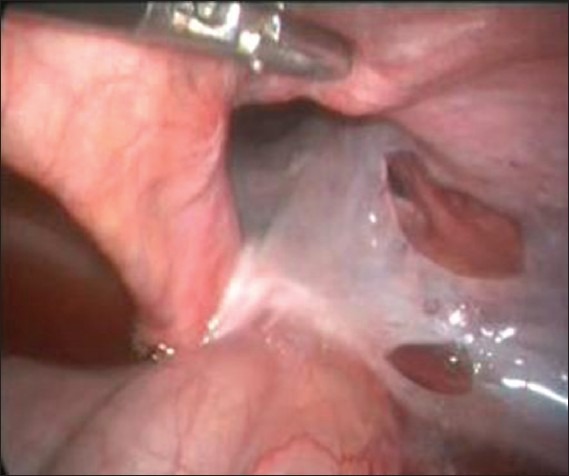
Deep ring is divided laterally and contents are reduced

All patients were operated upon under general anaesthesia. A pneumoperitoneum was created by a Veress needle inserted just below the umbilicus. One 10 mm and two 5 mm trocars were inserted. Working trocars were inserted in the midclavicular line on both sides. Hernias were indirect and on the right side in all four patients [Figure 1]. Laparoscopic examination revealed herniation of a small bowel in three patients and sliding hernia with herniation of ileocaecal junction in one patient. The incarcerated small bowel was return to the abdominal cavity with ease in two patients. The other two patients required the division of the internal ring to bring the hernia content into the abdominal cavity [Figure 2]. The internal ring was opened on the lateral side. All the patients had congested but viable bowel when examined after reduction. Resection of the bowel was not required in any patient. After reduction of the hernia contents, the hernia was repaired by raising the peritoneal flap. The sac was divided at the deep ring and the distal sac was left behind in all the patients. A 15 × 12 cm sized Prolene mesh was kept and peritoneal flap was sutured. The mean operative time was of 114.5 minutes (range: 98–130 min). The postoperative course was uneventful in three patients. One patient developed seroma formation that was treated conservatively. An average hospital stay was of 2.75 days (range: 2–4 days) [Table 1]. The mean follow-up period was of 3 months (range: 1–6 months) without any recurrence.

**Table 1:** Clinical and operative characteristics of patients operated with laparoscopic transperitoneal approach for irreducible inguinal hernias

Age	Sex	Symptoms	Duration of Symptoms (hours)	Contents	Division of deep ring	Duration of surgery (min)	Postop stay	Complications
26	M	Irreducibility; vomiting; abdominal distention	12	Small bowel	Yes	112	2	Nil
49	M	Irreducibility; abdominal distention	16	Small bowel	No	118	3	Nil
34	M	Irreducibility; abdominal distention	14	Sliding hernia	Yes	130	2	Nil
59	M	Irreducibility; vomiting; abdominal distention	13	Small bowel	No	98	4	Seroma

## DISCUSSION

Incarceration and strangulation are one of the most feared complications of the inguinal hernia. It is very important to release incarcerated hernia as soon as possible to avoid obstruction or strangulation of the bowel. Incarcerated hernias have generally been managed using the conventional technique of hernia repair.[7] It has been reported that postoperative complications were significantly more common after emergency treatment of incarcerated inguinal hernias then after elective repair of hernias in elderly.[10,11] One of the earliest successful repairs of incarcerated inguinal hernia with laparoscopy was reported by Watson in 1993.[8]

Since then few reports are available demonstrating feasibility and safety of laparoscopy in the management of incarcerated inguinal hernia.[7–9] Both approaches, total extraperitoneal (TEP) and transabdominal peritoneal (TAPP), have been described for the management of incarcerated inguinal hernia.

We prefer the TAPP approach for incarcerated hernia. There are several advantages of this approach over the TEP approach.

The TAPP approach is better in assessing the vitality of the intestine after the reduction of the hernia contents into the abdominal cavity.

After reduction of the bowel, it can be observe for a longer period of time that is not possible in the open and TEP approaches.[7,9] This is of great help when the vitality of the bowel is doubtful. The congested bowel can be observed till the completion of hernia repair, thus avoids unnecessary resection in the vital bowel and at the same time allows resection of the bowels that are not viable. Manipulation of the bowel should be very gentle during reduction. It is sometimes better to manipulate the bowel by holding the mesentery rather than bowel itself. This reduces the chances of inadvertent injury to the congested bowel. The reduced bowel should be inspected very carefully to detect serosal tear of the bowel. If the irreducible bowel is gangrenous, there are chances of peritonism after the reduction of the bowel. If there is any contamination, fluid should be sucked immediately and saline wash should be given. In total extraperitoneal repair, the assessment of the content is difficult if the sac is not opened. If the sac is opened to assess the viability of the bowel, there is a chance of inadvertent injury to the bowel.[12] In the TAPP approach, if the bowel is ischaemic, it is possible to deliver the bowel and resect it after putting a target incision just below the umbilicus.

It is not always possible to reduce the content of the incarcerated hernia without division of the internal ring as is seen in two of our cases. The division of the internal ring is often very difficult, as it is covered by oedematous bowel loops. The deep ring should be cut gently with scissors. If difficulty is encounter during division, a Maryland forceps or a grasper is inserted into the deep ring just lateral to the bowel and jaws of the instrument should be open. This will give some space to cut the ring safely. The division of the internal ring is under vision in the TAPP approach, which avoids injury to the bowel.

In few cases, the TAPP procedure also allows the discovery of additional intra-abdominal pathologies and their possible simultaneous treatment.[9] Moreover, the use of the TAPP technique facilitates inspection of the contralateral inguinal region and the discovery of unsuspected bilateral hernias. This allows repair of the contralateral hernia at the same time.

No attempt was made to dissect the whole sac from the cord structures. In all the patients, the sac was divided at the deep ring and the distal sac was left behind. Thus, less dissection of cord structures during the laparoscopic approach reduces the chances of cord oedema. If the problem of the recurrence after TAPP is considered, similar low rates can be found for repair of both reducible and incarcerated hernias.[9]

Nevertheless, laparoscopic hernia repair offers real advantages. These include a gain in diagnostic safety and the possibility it offers for assessing the vitality of incarcerated organs and for exploring additional intra-abdominal complications. Despite a little longer operating time for incarcerated hernias, data suggest that for incarcerated hernias, the TAPP approach is a safe and feasible option and should be considered for repair.

In conclusion, the laparoscopic approach is a feasible option for incarcerated inguinal hernia. The TAPP approach allows for the assessment of the bowel for a longer period of time than the TEP approach. The division of the internal ring can be done under vision without injuring the bowel. Undiagnosed inguinal hernia or any other intra-abdominal pathology can be diagnosed and treated at the same time in TAPP repair.
